# Identification and Isolation of Type II NKT Cell Subsets in Human Blood and Liver

**DOI:** 10.3389/fimmu.2022.898473

**Published:** 2022-06-02

**Authors:** Jordi Yang Zhou, Jens M. Werner, Gunther Glehr, Edward K. Geissler, James A. Hutchinson, Katharina Kronenberg

**Affiliations:** ^1^ Department of Surgery, University Hospital Regensburg, Regensburg, Germany; ^2^ Leibniz Institute for Immunotherapy, Regensburg, Germany; ^3^ Fraunhofer-Institute for Toxicology and Experimental Medicine Institute for Toxicology and Experimental Medicine-Regensburg (ITEM-R), Regensburg, Germany; ^4^ Regensburg International Graduate School of Life Sciences, University of Regensburg, Regensburg, Germany

**Keywords:** NKT, isolation, expansion, FoxP3, liver, steatotic

## Abstract

**Background:**

Steatotic livers are more prone to rejection, but are often transplanted owing to the shortage of available organs. Type II NKT (T2NKT) cells are liver-resident lymphocytes that react to lipids presented by CD1d. The role of T2NKT cells in rejection of fatty liver transplants is unclear, partly because of a lack of T2NKT cell markers and their very low frequency in blood. Here, we quantify human T2NKT cells in blood and liver tissue by flow cytometry and provide a strategy for their enrichment and expansion.

**Methods:**

Human T2NKT cells were identified as CD3^+^ CD56^+^ CD161^+^ TCR-γᵹ^-^ TCRVα7.2^-^ and TCRVα24^-^ cells. T2NKT cells were enriched from blood by sequential positive selection using CD56 and CD3 microbeads. These were subsequently FACS-sorted to purity then expanded *in vitro* for 3 weeks using anti-CD3/CD28 beads and TGF-β1.

**Results:**

The frequency of human T2NKT cells in blood was very low (0.8 ± 0.4% of CD3^+^ T cells) but they were a more abundant population in liver (6.3 ± 0.9%). Enriched T2NKT cells expressed the transcription factor PLZF. A novel subset of FoxP3^+^ T2NKT cells was discovered in blood and liver tissue. T2NKT cells were expanded in culture by 15- to 28-fold over 3 weeks, during which time they maintained expression of all identifying markers, including PLZF and FoxP3.

**Conclusions:**

Our work defines new strategies for identifying and isolating T2NKT cells from human blood and liver tissue. We showed that this rare population can be expanded *in vitro* in order to obtain experimentally amenable cell numbers. Further, we identified a novel T2NKT cell subset that stably expresses FoxP3, which might play a role in regulating innate-like lymphocyte responses in steatotic liver transplants.

## Introduction

Demand for liver transplants has significantly increased over the past decade (1). Approximately 25% of the global population is affected by nonalcoholic fatty liver disease, which is characterized by excessive accumulation of fat in hepatocytes, a condition known as steatosis. Thus, a high proportion of livers available for transplant are steatotic (2). Unfortunately, a significantly higher rate of primary graft dysfunction is observed after transplantation of steatotic organs (3). Moreover, moderate and severe steatosis of donor livers is associated with inferior post-transplant outcomes (4).

Natural Killer T (NKT) cells are non-conventional T cells that share common features with NK cells. NKT cells are rare in peripheral blood, but represent a larger fraction of liver, spleen, lung, and intestine resident T cells (5). Expression of T Cell Receptor (TCR)-αβ enables NKT cells to recognize antigenic lipids presented by CD1d, an MHC class I-like molecule (6). NKT cells express the promyelocytic leukemia zinc finger protein (PLZF) transcription factor as a prerequisite for their development (5, 7–9). Among T cells, only innate-like γᵹ T cells and MAIT cells otherwise express PLZF (5, 10). NKT cells are subclassified into invariant NKT (iNKT) cells and type II NKT (T2NKT) cells according to their TCR specificity. iNKT cells possess an invariant TCR Vα24-Jα18α chain paired with TCR Vβ11, which mainly responds to α-galactosylceramide glycopeptide (αGalCer). By contrast, T2NKT cells utilize different TCR rearrangements to recognize diverse lipids (11).

Our understanding of T2NKT cells in transplant immunology has been limited by the technical challenges of studying them, especially the very low frequency of these cells in blood and lack of exclusive markers. Human T2NKT cells are conventionally detected with sulfatide-loaded CD1d tetramers (12, 13); however, this method is noisy and fails to identify T2NKT cells that recognize other lipids. Here, we report a strategy to detect and isolate T2NKT cells from human blood and liver tissue. We further develop an expansion strategy to increase cell numbers, making it possible to conduct *in vitro* experiments.

## Methods

### Study Approval and Human Samples

Human peripheral blood leucocytes were collected from healthy individuals as a by-product of thrombocyte donation with the approval of the Ethics Committee of the University of Regensburg (approval number 19-1403-101). Intrahepatic lymphocytes (IHL) were isolated from resected liver tissues with the approval of the Ethics Committee of the University of Regensburg (approval number 13-257-101). All cell and tissue donors gave full, informed, written consent to the study.

### Isolation of Mononuclear Leucocytes From Apheresates and Liver

Peripheral blood mononuclear cells (PBMC) were isolated by Ficoll gradient centrifugation as previously described (14). IHL were isolated from surgically resected liver. Briefly, samples were washed with Hank’s balanced salt solution (HBSS) (H9394, Gibco, UK), minced with a sterile blade, and incubated in HBSS supplemented with 0.5 mg/ml collagenase type IV (C4-28-BC, Sigma-Aldrich, Germany) and 0.05 μg/ml DNase I (A37880, AppliChem, Germany) for 10 mins at 37°C. The suspension was filtered through a 100 µm nylon mesh filter, then through a 30 µm nylon mesh filter to remove cell clumps and hepatocytes. IHL were isolated by Ficoll gradient centrifugation and subsequently frozen in RPMI (72400-021, Gibco) with 10% DMSO at -160°C liquid nitrogen. For thawing, cryovials were placed in 37°C water bath for 1 min and then cells were transferred into a 15 ml Falcon tube with 1 ml prewarmed RPMI supplemented with 100 µg/ml DNase I. Cells were rested for 4 mins and incubated with 2 ml RPMI for 2 mins before adding 4 ml RPMI and incubating for 2 mins.

### Magnetic Bead Selection of T2NKT Cells

Starting with a single-cell suspension of PBMC or IHL, a two-step process was used to enrich T2NKT cells. In the first step, a typical starting number of 1 – 7 x 10^8^ cells were sorted with the REAlease CD56 MicroBead Kit (130-117-033, Miltenyi, Germany) according to the manufacturer’s instructions, using LS columns (130-042-401, Miltenyi) and Separation Buffer [PBS, 0.5% Albunorm (00200331, Octapharma, Germany), 0.4% UltraPure 0.5M EDTA (15575-038, Invitrogen, USA)]. In the second step, after “de-labelling” of CD56-selected cells, a second magnetic bead isolation was performed with CD3 MicroBeads (130-050-101, Miltenyi). Typically, 0.1 – 5 x 10^7^ recovered cells were positively selected according to the manufacturer’s instructions using the “Possel” selection routine on an AutoMACS Pro Separator (Miltenyi). After selection, enriched cells were resuspended in 2 ml NKT medium (500 ml RPMI Medium 1640 supplemented with 2 mM GlutaMAX™, 50 ml FCS, 5 ml Pen/Strep, 5 ml sodium pyruvate, 15 ml 7.5% NaHCO_3_, and 55 µl β-ME). Cells were then stored at 4°C overnight before further processing.

### Purification of T2NKT Cells by Flow Cytometry Sorting

Enriched cells were resuspended in 50 µl Cell Staining Buffer (CSB) (420201, Biolegend, USA) plus 10% FcR-Block (130-059-901, Miltenyi) then incubated for 15 min at 4°C. Cells were labelled with biotinylated antibodies against TCR-γᵹ (130-113-502, Miltenyi), TCR-Vα24 (130-117-958, Miltenyi) and TCR Vα7.2 (130-110-957, Miltenyi) in a total volume of 100 µl of CSB for 15 min at 4°C. After washing in 2 ml CSB, cells were stained for 30 min at 4°C with α-biotin FITC (130-110-957, Miltenyi), αCD161 PE (339904, BioLegend), αCD25 PC5.5 (356112, BioLegend), αCD56 APC (318310, BioLegend), αCD3 APC-H7 (560176, BD Biosciences, USA) and αCD4 BV421 (344632, BioLegend) in a final volume of 100 µl/sample comprising CSB plus 50 µl Brilliant Staining Buffer (563794, BD). Cells were washed with 2 ml CSB then resuspended in 500 µl PBS prior to sorting on a FACSAria Fusion (BD).

### Expansion of T2NKT Cells in Culture

On day 0, 0.5 – 1 x 10^5^ flow-sorted T2NKT cells were resuspended in 100 µl/well TexMACS medium (130-097-196, Miltenyi) supplemented with 500 U/ml recombinant human IL-2 (P30-2901, Peprotech, USA), and 5% human AB serum (P30-2901, PAN-Biotech, Germany) in U-bottom 96-well plates (3799, Costar, USA). Anti-CD3/CD28 MACSiBeads were prepared following the manufacturer’s instructions and added into the wells at a bead:cell ratio of 4:1. To promote expansion of FoxP3^+^ T2NKT cells, 5 pg/µl TGF-β1 (240-B-010/CF, R&D Systems, USA) was added to cultures. On day +1, wells were topped up with 100 µl TexMACS medium containing 5 pg/µl TGF-β1. Medium was changed every 3 days by replacing 100 µl consumed media with fresh TexMACS medium containing 5 pg/µl TGF-β1. After 2 weeks expansion, cells were harvested and resuspended in 1 ml TexMACS medium. A 10 µl aliquot of cells was counted using Precision Count Beads (424902, BioLegend). Remaining cells were maintained in culture for another 1 week with medium supplementation every 3 days.

### Identification of T2NKT Cells by Flow Cytometry

Samples were prepared for analysis by flow cytometry according to previously published methods (15). Briefly, 10^6^ cells were transferred into V-bottom 96 well microplates (651201, Greiner, Germany) with 50 µl/well CSB plus 10% FcR-Block for 15 min at 4°C. Biotin-labelled antibodies against TCR-Vα24, TCR-vα7.2, and TCR-γᵹ were added and adjusted with CSB to 100 µl/well. Samples were incubated for 15 min at 4°C and washed with 150 µl/well CSB. Samples were resuspended in 50 µl Brilliant Staining Buffer with following antibodies and adjusted to a total volume of 100 µl with CSB: α-CD161 PE (339904, Biolegend); α-CD25 PC5.5 (356112, BioLegend); α-CD127 BV421 (351332, BioLegend); α-CD56 BV510 (318340, BioLegend); α-Biotin FITC (130-110-957, Miltenyi); CD3 APC-H7 (560176, BD). Samples were incubated for 30 min at 4°C then washed twice with 200 µl/well CSB. Cells were then stained for intracellular markers using the eBioscience FOXP3/Transcription Factor Staining Buffer Set (00-5523-00, Invitrogen, USA) according to the manufacturer’s instructions. After fixation and permeabilization, samples were stained with α-PLZF PC7 (25-9322-82, Invitrogen, Germany) and α-FoxP3 (320213, Biolegend) for 30 mins at 4°C. Cells were washed in CSB then resuspended in 1X Fixative Solution IOTest3 solution (A07800, Beckman Coulter, Germany) prior to measurement with a Canto-II (BD) or CytoFlex LX (Beckman Coulter) cytometer.

Sulfatide-loaded CD1d tetramers were prepared as previously described (12). Briefly, 1.7 µg sulfatide (1049, Matreya) were incubated with 11 µg human CD1d Unloaded Biotinylated Monomer (kindly provided by the NIH Tetramer Core Facility) in a total volume of 20 µl PBS at 37°C overnight. The next day, 1 µl, 10 µl, or 100 µl of 1:10 PE streptavidin (405203, Biolegend) in sterile PBS was mixed with the complex and incubated for 2 h at RT in a total volume of 110 µl. 10^6^ PBMC or IHL were incubated with 50 µl/well PBS plus 10% FcR-Block in V-bottom 96 well microplates for 15 mins at 4°C. Cells were then stained with 1 µl, 5 µl or 10 µl of each sulfatide-CD1d preparation with gentle mixing and incubated for 30 mins at 4°C. Cells were then stained for flow cytometry as described above without an intervening washing step.

## Results

### Establishing a Flow Cytometry Panel to Quantify T2NKT Cells

**Figure 1:**
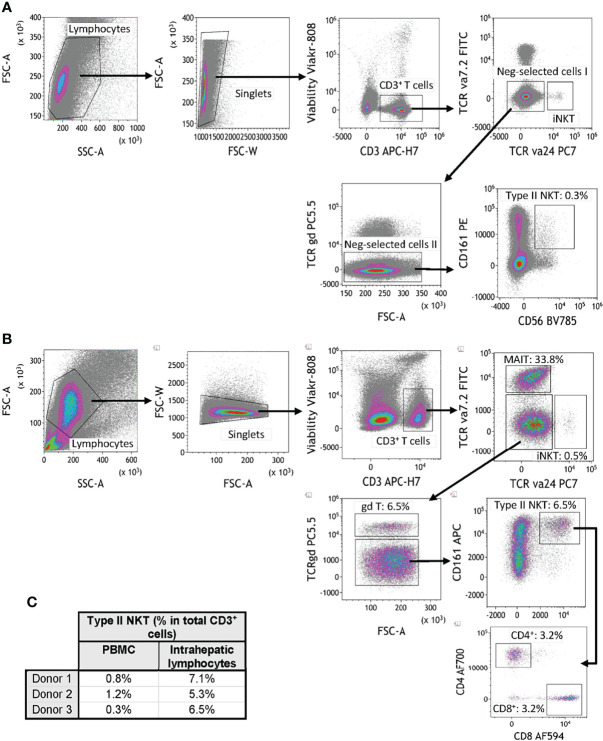
Flow cytometry gating strategy for quantification of human type II NKT cells. **(A)** Human T2NKT cells were defined as CD3^+^ CD56^+^ CD161^+^ TCRγδ^-^ TCRVα7.2^-^ TCRVα24^-^ lymphocytes. Gating of human PBMC resulted in a very low frequency of circulating T2NKT cells (0.3% of total CD3^+^ T cells). **(B)** Gating of human intrahepatic lymphocytes (IHL) revealed a higher frequency of T2NKT cells in non-steatotic liver (6.5% of total CD3^+^ T cells). Intrahepatic T2NKT cells could be subdivided into CD4^+^ and CD8^+^ cells. **(C)** Frequency of human T2NKT cells with respect to CD3^+^ T cells from 3 PBMC and 3 liver samples (steatosis 0%, n = 2; steatosis 15%, n = 1).

T2NKT cells are challenging to study because of a lack of positive markers and their very low frequency in blood (5, 8). Although NKT cells can be positively identified using CD1d tetramers loaded with lipids (such as α-GalCer, sulfatide and lysosulfatides) this is neither a convenient nor reliable approach for quantifying total T2NKT cells in a single sample (8, 16) (**Supplementary Figure 1**). Therefore, we propose a working definition of T2NKT cells as CD3^+^ CD56^+^ CD161^+^ T cells after excluding TCR-Vα24^+^ iNKT cells, TCR-γᵹ^+^ T cells and TCR-Vα7.2^+^ MAIT cells (**Figures 1A, B**). It is useful to further subdivide T2NKT cells into CD4^+^, CD8^+^ and double-negative (DN) subpopulations, which may reflect functional differences in cytokine production (17). Our panel also included two members of the NKG2 (CD159) C-type lectin-like receptor family that are commonly expressed in NK cells and other innate-like lymphocytes (16, 18). CD4^-^ iNKT cells reportedly express NKG2D, enabling them to respond to MHC Class I-like molecules (*eg*. H60, RAE, MULT1 and MIC families) (19). By contrast, NKG2A has an inhibitory function. We anticipate that NKG2A and NKG2D will be useful markers of T2NKT cell activation in future immune monitoring studies. To improve the accuracy of our cell type identification, particularly when also using CD1d tetramers, we included CD16 and CD19 to exclude monocytes and B cells (20). Using this assay panel, we identified a CD3^+^ CD56^+^ CD161^+^ TCR-Vα24^-^ TCR-γᵹ^-^ TCR-Vα7.2^-^ living T2NKT cell population that represented 0.8 ± 0.4% of CD3^+^ T cells from PBMC and 6.3 ± 0.9% of CD3^+^ T cells from IHL preparations (**Figure 1C**).

### Pre-Enriching CD3^+^ CD56^+^ NKT Cells by Positive Selection

**Figure 2:**
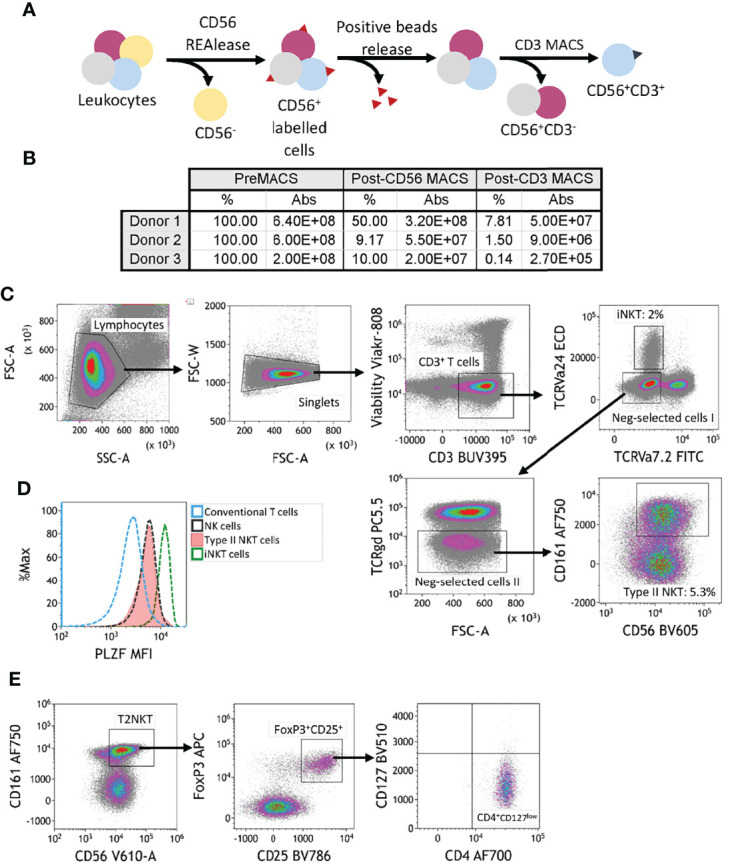
Two-step magnetic-activated cell sorting (MACS) strategy for enrichment of human T2NKT cells. ***(*A*)*** Two-step MACS strategy consisted first in a CD56 positive selection from human PBMC with CD56 REAlease microbeads. These beads were then detached from the cell surface after separation. In the second step, NKT cells were enriched using CD3 microbeads. The outcome of this procedure was a CD3^+^ CD56^+^ cell isolate enriched in NKT cells. **(B)** Summary table indicating the frequency of cells relative to preMACS values and absolute (Abs) cell numbers after each MACS step. **(C)** Gating of human leucocytes enriched for CD56^+^ CD3^+^ cells using the two-step MACS protocol. The sample was enriched for iNKT cells (2% of total CD3^+^ T cells) and T2NKT cells (5.3% of total CD3^+^ T cells). **(D)** Expression of the transcription regulator promyelocytic leukemia zinc finger (PLZF) in subsets of human lymphocytes. Differential expression of PLZF was observed between conventional T cells (blue line) and NK cells (black line). There was similar PLZF expression in T2NKT cells (red line) and NK cells, whereas iNKT cells (green line) showed higher expression. **(E)** A CD4^+^ FoxP3^+^ CD25^+^ CD127^low^ subpopulation of CD3^+^ CD56^+^ CD161^+^ TCRγδ^-^ TCRVα7.2^-^ TCRVα24^-^ T2NKT cells after the two-step enrichment process was identified in all 3 donors (one donor is shown as an example).

Because circulating T2NKT cells are so rare, we next developed a magnetic bead-based protocol to pre-enrich CD3^+^ CD56^+^ innate lymphocytes prior to immunophenotyping by flow cytometry or further purification by flow-sorting **(** **Figure 2A** **)**. We compared alternative separation strategies (**Supplementary Figures 2, 3**), namely: (1) Sorting for CD3^+^ cells then CD56^+^ cells; (2) sorting for CD56^+^ cells then CD3^+^ cells; (3) sorting using a CD3^+^ CD56^+^ NKT Cell Isolation Kit from Miltenyi Biotec; (4) sorting for CD56^+^ cells, then CD3^+^ cells and then depleting TCR-Vα24^+^ iNKT cells, TCR-γᵹ^+^ T cells and TCR-Vα7.2^+^ MAIT cells; (5) sorting for CD56^+^ cells, then CD3^+^ cells, then depleting TCR-Vα24^+^ iNKT cells, TCR-γᵹ^+^ T cells and TCR-Vα7.2^+^ MAIT cells and then enriching for CD161^+^ cells. We found that enriching CD3^+^ cells prior to CD56^+^ selection (Strategy 1) led to poorer yields than first enriching CD56^+^ cells (Strategy 2) (**Supplementary Figures 2A–C**). Miltenyi’s CD3^+^ CD56^+^ NKT Cell Isolation Kit (Strategy 3) performed well in enriching CD3^+^ CD56^+^ NKT cells, but led to a disproportionate loss of T2NKT compared to iNKT cells **(** **Supplementary Figure 3A** **)**. We attempted to increase the purity of our enriched samples by depleting TCR-Vα24^+^ iNKT cells, TCR-γᵹ^+^ T cells and TCR-Vα7.2^+^ MAIT cells without (Strategy 4) or with (Strategy 5) positive selection of CD161^+^ cells. Both methods had unacceptably low yields **(** **Supplementary Figures 3B, C** **)**. Hence, we determined that enriching CD56^+^ cells using REAlease CD56 MicroBead Kit from Miltenyi prior to positive selection of CD3^+^ cells using CD3 MicroBeads from Miltenyi is an optimally efficient method for pre-enriching human T2NKT cells from blood **(** **Figures 2B, C** **)**.

### Expression of PLZF and FoxP3 in CD3^+^ CD56^+^ CD161^+^ T2NKT Cells

The transcription factor PLZF is primarily expressed by innate-like lymphocytes, especially NK cells, MAIT cells and NKT cells. Intracellular staining of pre-enriched NKT cells from blood revealed an intermediate level of PLZF in T2NKT cells, which was similar to PLZF expression in NK cells, but less than PLZF expression in iNKT cells and greater than PLZF expression in conventional T cells (**Figure 2D**).

We hypothesize that distinct subsets of human T2NKT cells play inflammatory or regulatory roles in steatotic livers after transplantation; therefore, we next investigated expression of Treg-associated markers in pre-enriched NKT cells. This revealed a previously undescribed subset of CD3^+^ CD56^+^ CD161^+^ CD4^+^ CD25^+^ CD127^low^ PLZF^+^ FoxP3^+^ T2NKT cells that represented 4.8 ± 1.3% (n=3) of T2NKT cells from PBMC (**Figure 2E**). Additionally, among other innate-like lymphocytes, we identified a FoxP3^+^ MAIT cell population, but not a FoxP3^+^ iNKT cell or γᵹ T cell population (**Supplementary Figure 4**).

### Expansion of T2NKT Cells Without Losing NKT Cell Markers

**Figure 3:**
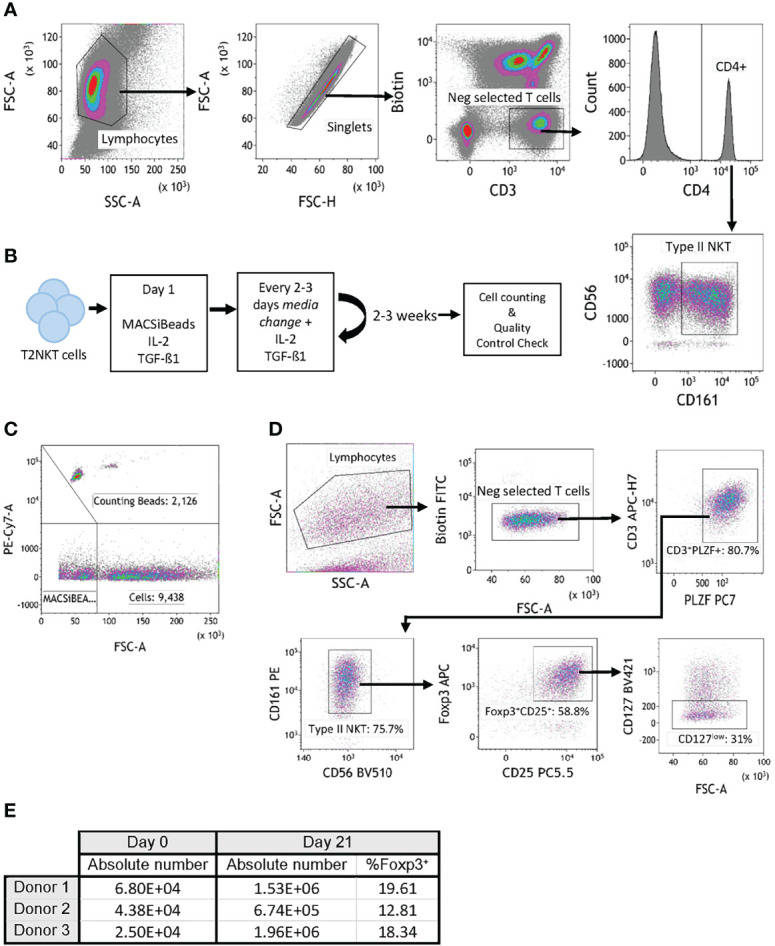
In vitro expansion of FoxP3^+^ T2NKT cells. **(A)** Gating strategy to flow-sort T2NKT cell after double-step MACS enrichment. From left to right, cells were gated for lymphocytes based on FSC/SSC and singlets. Biotin FITC excluded cells labeled with TCRVα7.2, TCRVα24 or TCRγᵹ antibody. Cells were then gated for CD3^+^ CD4^+^ CD56^+^ CD161^+^. **(B)** Schematic map depicting each step of the expansion protocol. **(C)** Cell counting after 3 weeks expansion using Flow Cytometry Counting Beads. Counting beads were positive for PE-Cy7, cells were separated from MACSiBeads based on FSC. **(D)** Quality control analysis of T2NKT cell expansion after 3 weeks. Lymphocytes were separated from MACSiBeads and dead cells by FCS/SSC. MAIT cells, γᵹ T cells and iNKT cells were excluded by staining with Biotin FITC. Within the CD3^+^ T cell population, PLZF was highly expressed (80.7% of the total lymphocyte population). These cells were mainly CD56^+^ CD161^+^ (75.7% of the total lymphocyte population) and a subset expressed regulatory-like phenotype FoxP3^+^ CD25^+^ CD127^low^ (31% of the total lymphocyte population). **(E)** Summary table indicating absolute number of cells before expansion (day 0) and after expansion (day 21). Percentage of FoxP3^+^ cells after expansion is included.

To perform functional experiments with T2NKT cells, it was necessary to flow-sort them to purity. NKT cells were enriched from PBMC by magnetic bead selection then stained with CD3, CD4, CD56, CD161, TCR-Vα24, TCR-Vα7.2 and TCR-γᵹ for flow-sorting (**Supplementary Figure 5**). As illustrated, T2NKT cells were isolated as CD3^+^ CD4^+^ CD56^+^ CD161^+^ TCR-Vα24^-^ TCR-Vα7.2^-^ and TCR-γᵹ^-^ living cells (**Figure 3A**). Following this strategy, 2 – 7 x 10^4^ T2NKT cells could be isolated from a typical starting population of 4 – 6 x 10^8^ unsorted PBMC.

Because the absolute number of T2NKT cells recovered with our optimized protocol was so limited, we next investigated whether they could be expanded *in vitro*. Purified T2NKT cells were stimulated with MACSiBeads, IL-2 and TGF-β1 for 3 weeks (**Figure 3B**). The cultured cells were then counted by flow cytometry (**Figure 3C**). After 21 days of *in vitro* stimulation, T2NKT cells maintained expression of CD3^+^ CD56^+^ CD161^+^ and PLZF **(** **Figure 3D** **)**. In addition, a subset of CD3^+^ CD56^+^ CD161^+^ CD4^+^ CD25^+^ CD127^low^ PLZF^+^ FoxP3^+^ T2NKT cells was still detectable, representing 16.9 ± 3.6% of total T2NKT cells (**Figures 3D, E**). Hence, we were able to expand T2NKT cells by ≥15-fold without loss of key markers, meaning that we can obtain sensible numbers of phenotypically normal T2NKT cells for use in functional studies.

## Discussion

Here, we present a strategy for identifying and enriching human T2NKT cells from blood and liver tissue. Further, we describe a novel subset of T2NKT cells that stably expresses FoxP3, suggesting it may play a role in regulating innate-like lymphocyte responses in fatty livers.

We propose a working definition of T2NKT cells based upon CD3, CD56 and CD161 coexpression, and exclusion of iNKT cells, MAIT cells and γᵹ T cells. Practically speaking, our definition is superior to detection with lipid-loaded CD1d tetramers for several reasons - namely, lipid-loaded CD1d tetramers label only a subset of T2NKT cells at best, CD1d tetramer staining is variable and does not clearly resolve positive and negative populations, and we lack a positive-control cell. We corroborated our working definition of T2NKT cells by showing an intermediate level of PLZF expression in the population identified as T2NKT cells (21).

Our optimized sorting protocol achieves an overall recovery of 2.5 – 6.8 x 10^4^ T2NKT cells from a starting number of 2 – 7 x 10^8^ peripheral blood mononuclear cells, which is an acceptable yield for such a rare cell population. Although the absolute number of T2NKT cells recovered was very low, we were able to expand them in culture by more than 15-fold without detectable phenotypic changes. Therefore, we can reliably obtain sufficient T2NKT cells for *in vitro* functional experiments.

This short report provides a technical basis for future immune monitoring studies. In particular, our methods will allow us to investigate how the balance between “effector” FoxP3^-^ T2NKT cells and putative “regulatory” FoxP3^+^ T2NKT cells influence outcomes after transplantation of steatotic livers.

## Data Availability Statement

The raw data supporting the conclusions of this article will be made available by the authors, without undue reservation.

## Ethics Statement

The studies involving human participants were reviewed and approved by Ethics Committee University of Regensburg. The patients/participants provided their written informed consent to participate in this study.

## Author Contributions

JY conceived the project, designed and performed experiments, analyzed data and wrote the manuscript. JW analyzed data and approved the manuscript. GG provided statistical review. EG approved the manuscript. JH provided critical feedback and edited the manuscript. KK edited the manuscript and is corresponding author. All authors contributed to the article and approved the submitted version.

## Conflict of Interest

The authors declare that the research was conducted in the absence of any commercial or financial relationships that could be construed as a potential conflict of interest.

## Publisher’s Note

All claims expressed in this article are solely those of the authors and do not necessarily represent those of their affiliated organizations, or those of the publisher, the editors and the reviewers. Any product that may be evaluated in this article, or claim that may be made by its manufacturer, is not guaranteed or endorsed by the publisher.
