# Supplementary material for: Identification and Isolation of Type II NKT Cell Subsets in Human Blood and Liver
Source: Front Immunol. 2022 Jun 2;13:898473. doi: 10.3389/fimmu.2022.898473 (PMC9202826; doi:10.3389/fimmu.2022.898473)
Supplement: Supplementary file 1 [file Presentation_1.pptx]

## Slide 1
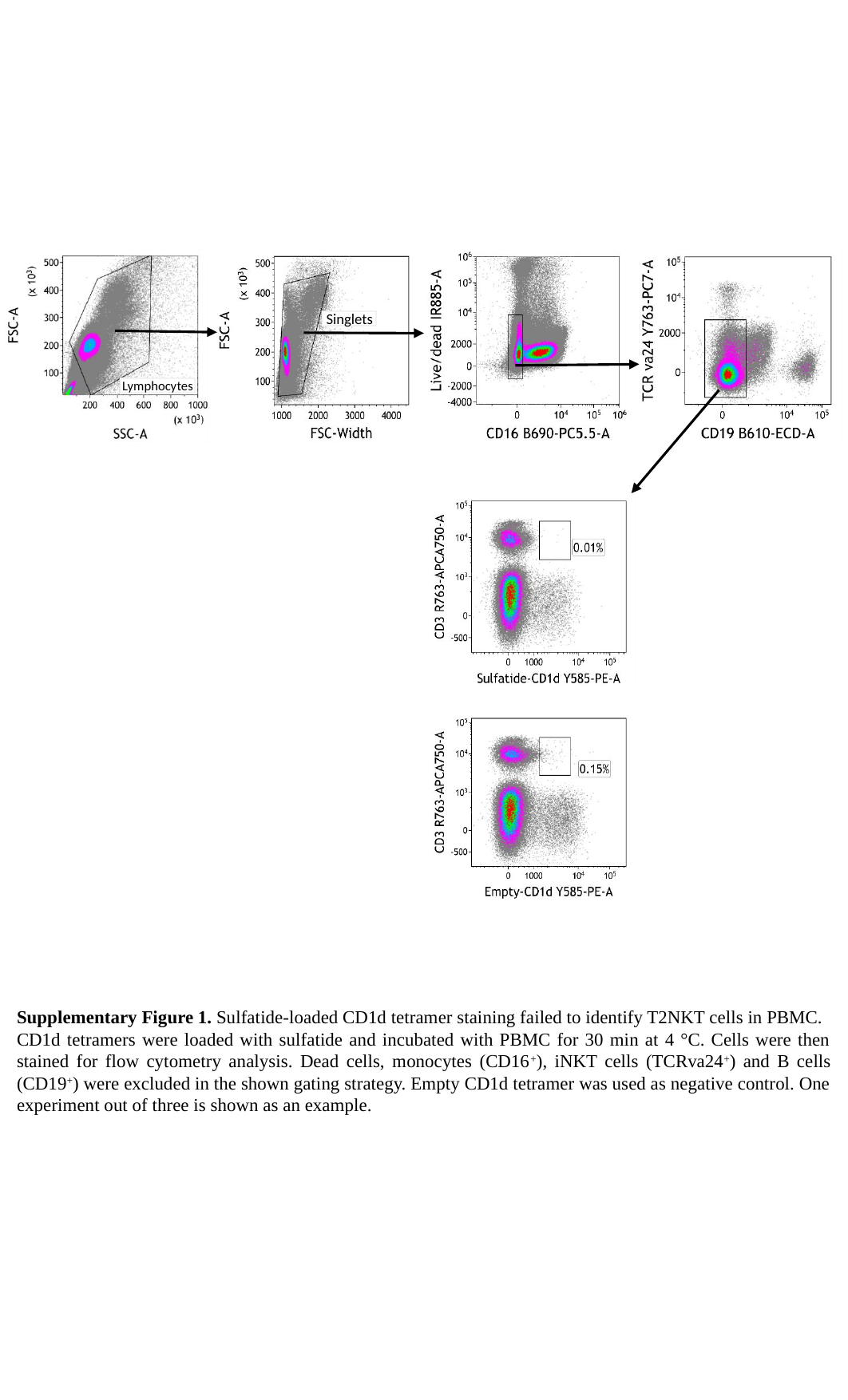

Singlets
Lymphocytes
Supplementary Figure 1. Sulfatide-loaded CD1d tetramer staining failed to identify T2NKT cells in PBMC.
CD1d tetramers were loaded with sulfatide and incubated with PBMC for 30 min at 4 °C. Cells were then stained for flow cytometry analysis. Dead cells, monocytes (CD16+), iNKT cells (TCRva24+) and B cells (CD19+) were excluded in the shown gating strategy. Empty CD1d tetramer was used as negative control. One experiment out of three is shown as an example.

## Slide 2
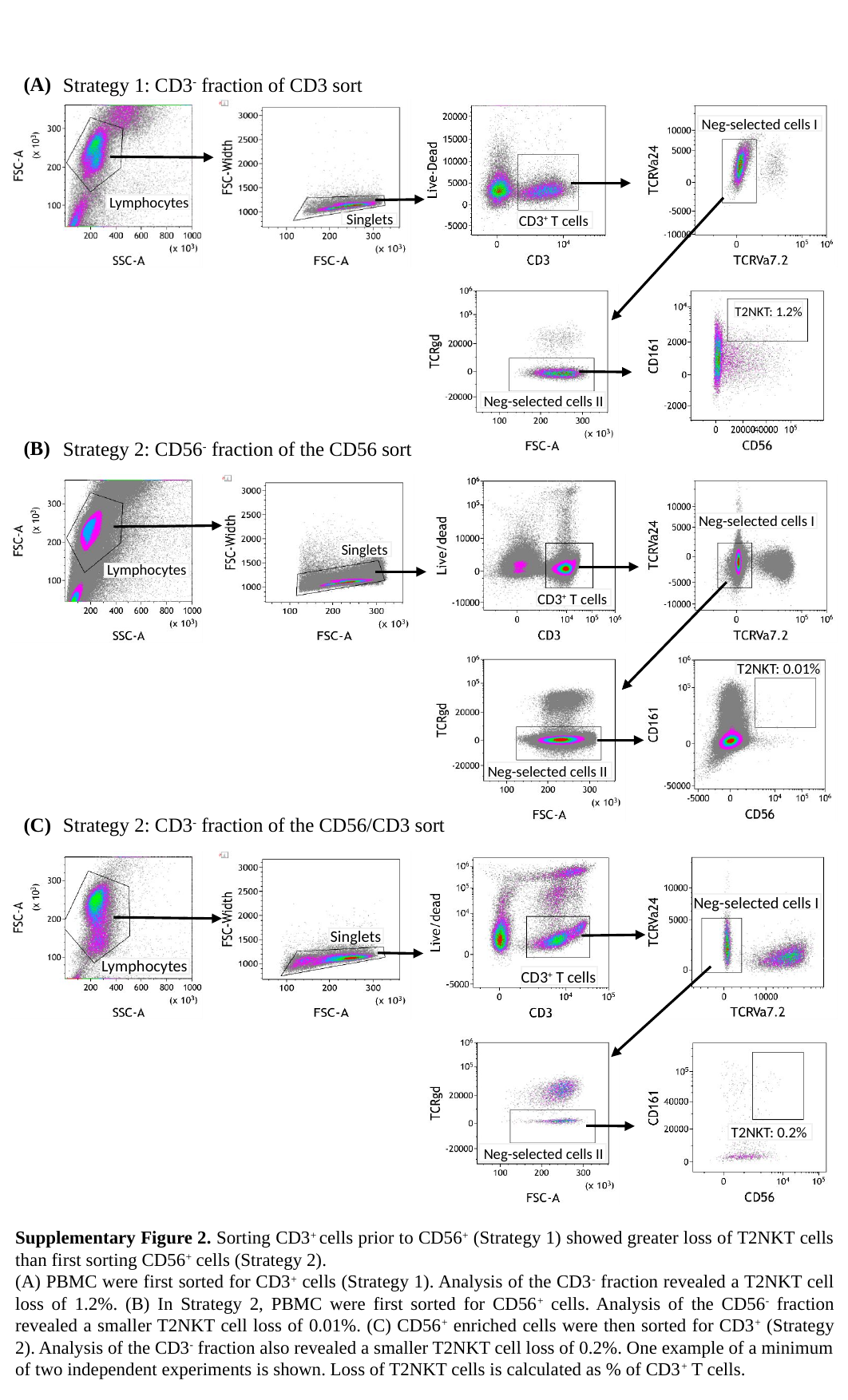

(A)
Strategy 1: CD3- fraction of CD3 sort
Neg-selected cells I
Lymphocytes
Singlets
CD3+ T cells
T2NKT: 1.2%
Neg-selected cells II
(B)
Strategy 2: CD56- fraction of the CD56 sort
Neg-selected cells I
Singlets
Lymphocytes
CD3+ T cells
T2NKT: 0.01%
Neg-selected cells II
(C)
Strategy 2: CD3- fraction of the CD56/CD3 sort
Neg-selected cells I
Singlets
Lymphocytes
CD3+ T cells
T2NKT: 0.2%
Neg-selected cells II
Supplementary Figure 2. Sorting CD3+ cells prior to CD56+ (Strategy 1) showed greater loss of T2NKT cells than first sorting CD56+ cells (Strategy 2).
(A) PBMC were first sorted for CD3+ cells (Strategy 1). Analysis of the CD3- fraction revealed a T2NKT cell loss of 1.2%. (B) In Strategy 2, PBMC were first sorted for CD56+ cells. Analysis of the CD56- fraction revealed a smaller T2NKT cell loss of 0.01%. (C) CD56+ enriched cells were then sorted for CD3+ (Strategy 2). Analysis of the CD3- fraction also revealed a smaller T2NKT cell loss of 0.2%. One example of a minimum of two independent experiments is shown. Loss of T2NKT cells is calculated as % of CD3+ T cells.

## Slide 3
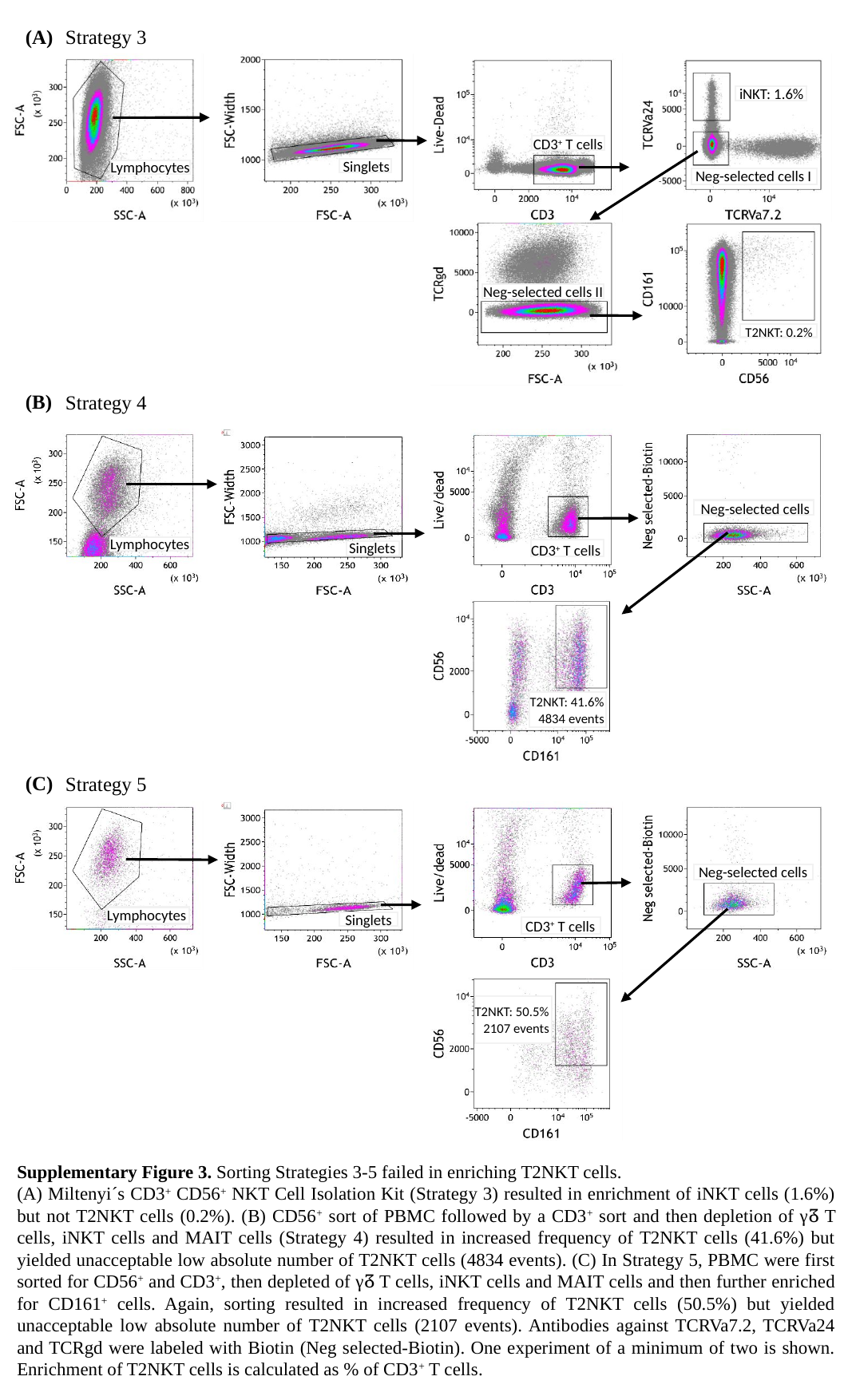

(A)
Strategy 3
iNKT: 1.6%
CD3+ T cells
Singlets
Lymphocytes
Neg-selected cells I
Neg-selected cells II
T2NKT: 0.2%
(B)
Strategy 4
Neg-selected cells
Lymphocytes
Singlets
CD3+ T cells
T2NKT: 41.6% 4834 events
(C)
Strategy 5
Neg-selected cells
Lymphocytes
Singlets
CD3+ T cells
T2NKT: 50.5% 2107 events
Supplementary Figure 3. Sorting Strategies 3-5 failed in enriching T2NKT cells.
(A) Miltenyi´s CD3+ CD56+ NKT Cell Isolation Kit (Strategy 3) resulted in enrichment of iNKT cells (1.6%) but not T2NKT cells (0.2%). (B) CD56+ sort of PBMC followed by a CD3+ sort and then depletion of γꝽ T cells, iNKT cells and MAIT cells (Strategy 4) resulted in increased frequency of T2NKT cells (41.6%) but yielded unacceptable low absolute number of T2NKT cells (4834 events). (C) In Strategy 5, PBMC were first sorted for CD56+ and CD3+, then depleted of γꝽ T cells, iNKT cells and MAIT cells and then further enriched for CD161+ cells. Again, sorting resulted in increased frequency of T2NKT cells (50.5%) but yielded unacceptable low absolute number of T2NKT cells (2107 events). Antibodies against TCRVa7.2, TCRVa24 and TCRgd were labeled with Biotin (Neg selected-Biotin). One experiment of a minimum of two is shown. Enrichment of T2NKT cells is calculated as % of CD3+ T cells.

## Slide 4
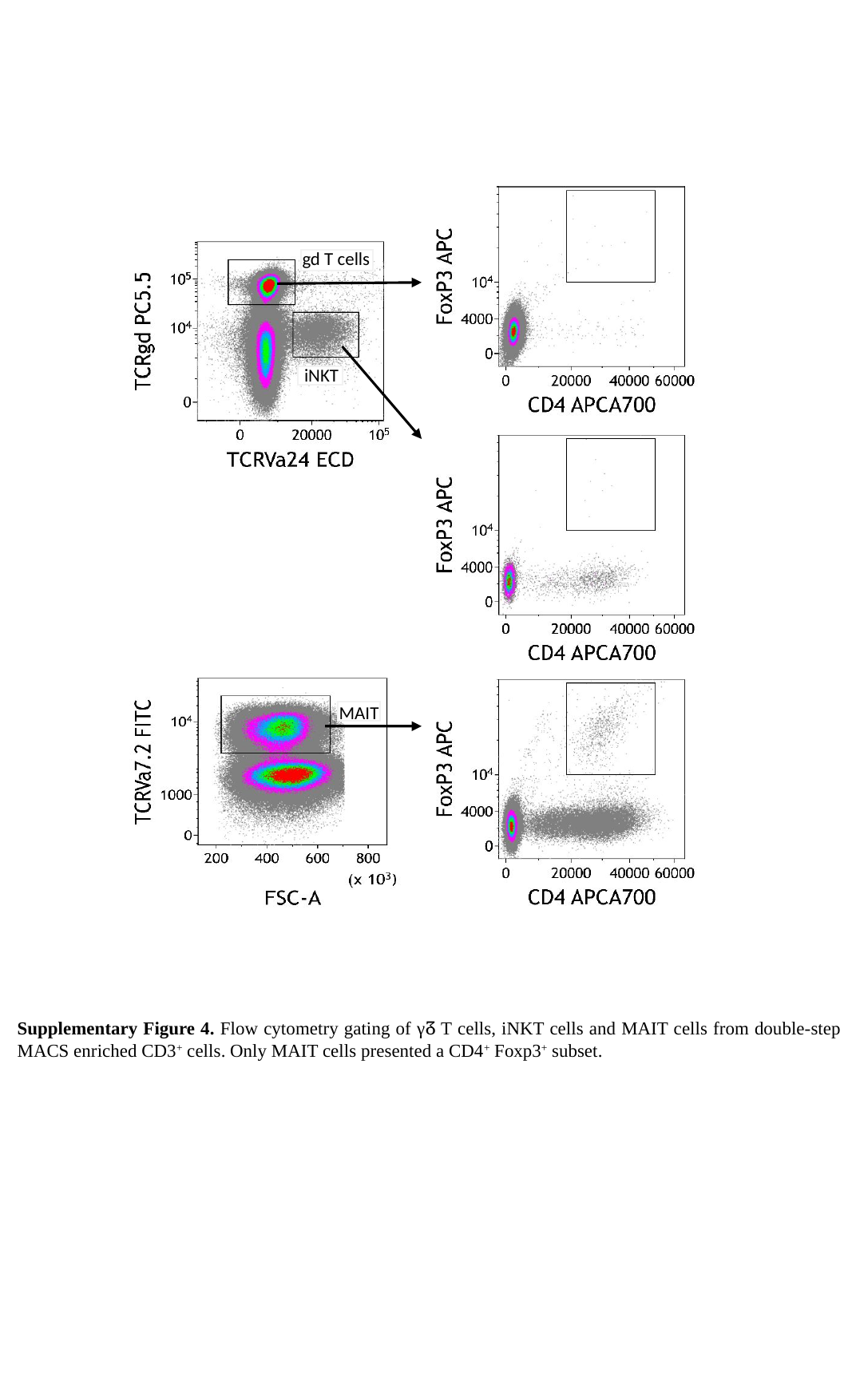

gd T cells
iNKT
MAIT
Supplementary Figure 4. Flow cytometry gating of γꝽ T cells, iNKT cells and MAIT cells from double-step MACS enriched CD3+ cells. Only MAIT cells presented a CD4+ Foxp3+ subset.

## Slide 5
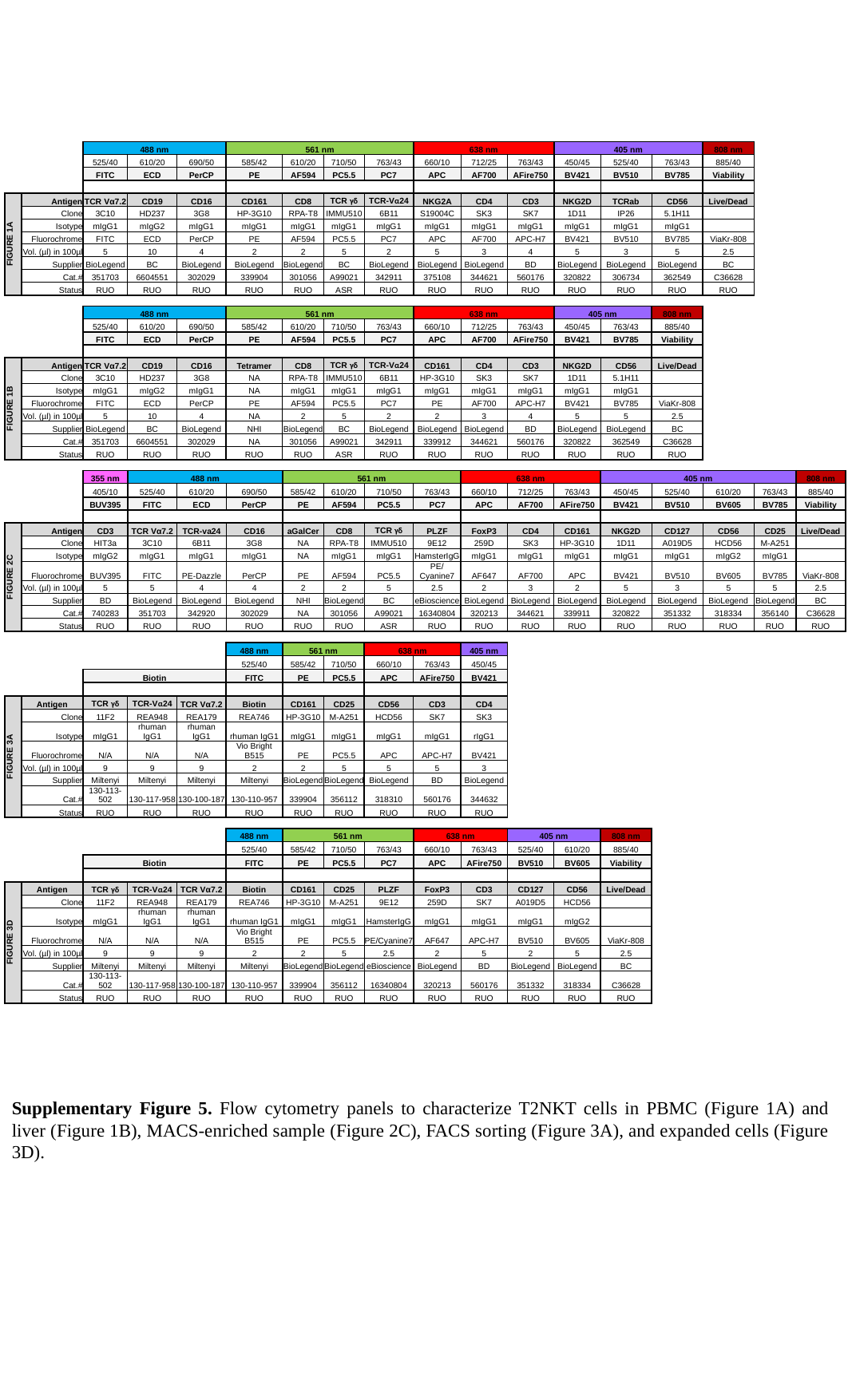

| | | 488 nm | | | 561 nm | | | | 638 nm | | | 405 nm | | | 808 nm | | |
| --- | --- | --- | --- | --- | --- | --- | --- | --- | --- | --- | --- | --- | --- | --- | --- | --- | --- |
| | | 525/40 | 610/20 | 690/50 | 585/42 | 610/20 | 710/50 | 763/43 | 660/10 | 712/25 | 763/43 | 450/45 | 525/40 | 763/43 | 885/40 | | |
| | | FITC | ECD | PerCP | PE | AF594 | PC5.5 | PC7 | APC | AF700 | AFire750 | BV421 | BV510 | BV785 | Viability | | |
| | | | | | | | | | | | | | | | | | |
| FIGURE 1A | Antigen | TCR Vα7.2 | CD19 | CD16 | CD161 | CD8 | TCR γδ | TCR-Vα24 | NKG2A | CD4 | CD3 | NKG2D | TCRab | CD56 | Live/Dead | | |
| | Clone | 3C10 | HD237 | 3G8 | HP-3G10 | RPA-T8 | IMMU510 | 6B11 | S19004C | SK3 | SK7 | 1D11 | IP26 | 5.1H11 | | | |
| | Isotype | mIgG1 | mIgG2 | mIgG1 | mIgG1 | mIgG1 | mIgG1 | mIgG1 | mIgG1 | mIgG1 | mIgG1 | mIgG1 | mIgG1 | mIgG1 | | | |
| | Fluorochrome | FITC | ECD | PerCP | PE | AF594 | PC5.5 | PC7 | APC | AF700 | APC-H7 | BV421 | BV510 | BV785 | ViaKr-808 | | |
| | Vol. (µl) in 100µl | 5 | 10 | 4 | 2 | 2 | 5 | 2 | 5 | 3 | 4 | 5 | 3 | 5 | 2.5 | | |
| | Supplier | BioLegend | BC | BioLegend | BioLegend | BioLegend | BC | BioLegend | BioLegend | BioLegend | BD | BioLegend | BioLegend | BioLegend | BC | | |
| | Cat.# | 351703 | 6604551 | 302029 | 339904 | 301056 | A99021 | 342911 | 375108 | 344621 | 560176 | 320822 | 306734 | 362549 | C36628 | | |
| | Status | RUO | RUO | RUO | RUO | RUO | ASR | RUO | RUO | RUO | RUO | RUO | RUO | RUO | RUO | | |
| | | | | | | | | | | | | | | | | | |
| | | 488 nm | | | 561 nm | | | | 638 nm | | | 405 nm | | 808 nm | | | |
| | | 525/40 | 610/20 | 690/50 | 585/42 | 610/20 | 710/50 | 763/43 | 660/10 | 712/25 | 763/43 | 450/45 | 763/43 | 885/40 | | | |
| | | FITC | ECD | PerCP | PE | AF594 | PC5.5 | PC7 | APC | AF700 | AFire750 | BV421 | BV785 | Viability | | | |
| | | | | | | | | | | | | | | | | | |
| FIGURE 1B | Antigen | TCR Vα7.2 | CD19 | CD16 | Tetramer | CD8 | TCR γδ | TCR-Vα24 | CD161 | CD4 | CD3 | NKG2D | CD56 | Live/Dead | | | |
| | Clone | 3C10 | HD237 | 3G8 | NA | RPA-T8 | IMMU510 | 6B11 | HP-3G10 | SK3 | SK7 | 1D11 | 5.1H11 | | | | |
| | Isotype | mIgG1 | mIgG2 | mIgG1 | NA | mIgG1 | mIgG1 | mIgG1 | mIgG1 | mIgG1 | mIgG1 | mIgG1 | mIgG1 | | | | |
| | Fluorochrome | FITC | ECD | PerCP | PE | AF594 | PC5.5 | PC7 | PE | AF700 | APC-H7 | BV421 | BV785 | ViaKr-808 | | | |
| | Vol. (µl) in 100µl | 5 | 10 | 4 | NA | 2 | 5 | 2 | 2 | 3 | 4 | 5 | 5 | 2.5 | | | |
| | Supplier | BioLegend | BC | BioLegend | NHI | BioLegend | BC | BioLegend | BioLegend | BioLegend | BD | BioLegend | BioLegend | BC | | | |
| | Cat.# | 351703 | 6604551 | 302029 | NA | 301056 | A99021 | 342911 | 339912 | 344621 | 560176 | 320822 | 362549 | C36628 | | | |
| | Status | RUO | RUO | RUO | RUO | RUO | ASR | RUO | RUO | RUO | RUO | RUO | RUO | RUO | | | |
| | | | | | | | | | | | | | | | | | |
| | | 355 nm | 488 nm | | | 561 nm | | | | 638 nm | | | 405 nm | | | | 808 nm |
| | | 405/10 | 525/40 | 610/20 | 690/50 | 585/42 | 610/20 | 710/50 | 763/43 | 660/10 | 712/25 | 763/43 | 450/45 | 525/40 | 610/20 | 763/43 | 885/40 |
| | | BUV395 | FITC | ECD | PerCP | PE | AF594 | PC5.5 | PC7 | APC | AF700 | AFire750 | BV421 | BV510 | BV605 | BV785 | Viability |
| | | | | | | | | | | | | | | | | | |
| FIGURE 2C | Antigen | CD3 | TCR Vα7.2 | TCR-va24 | CD16 | aGalCer | CD8 | TCR γδ | PLZF | FoxP3 | CD4 | CD161 | NKG2D | CD127 | CD56 | CD25 | Live/Dead |
| | Clone | HIT3a | 3C10 | 6B11 | 3G8 | NA | RPA-T8 | IMMU510 | 9E12 | 259D | SK3 | HP-3G10 | 1D11 | A019D5 | HCD56 | M-A251 | |
| | Isotype | mIgG2 | mIgG1 | mIgG1 | mIgG1 | NA | mIgG1 | mIgG1 | HamsterIgG | mIgG1 | mIgG1 | mIgG1 | mIgG1 | mIgG1 | mIgG2 | mIgG1 | |
| | Fluorochrome | BUV395 | FITC | PE-Dazzle | PerCP | PE | AF594 | PC5.5 | PE/Cyanine7 | AF647 | AF700 | APC | BV421 | BV510 | BV605 | BV785 | ViaKr-808 |
| | Vol. (µl) in 100µl | 5 | 5 | 4 | 4 | 2 | 2 | 5 | 2.5 | 2 | 3 | 2 | 5 | 3 | 5 | 5 | 2.5 |
| | Supplier | BD | BioLegend | BioLegend | BioLegend | NHI | BioLegend | BC | eBioscience | BioLegend | BioLegend | BioLegend | BioLegend | BioLegend | BioLegend | BioLegend | BC |
| | Cat.# | 740283 | 351703 | 342920 | 302029 | NA | 301056 | A99021 | 16340804 | 320213 | 344621 | 339911 | 320822 | 351332 | 318334 | 356140 | C36628 |
| | Status | RUO | RUO | RUO | RUO | RUO | RUO | ASR | RUO | RUO | RUO | RUO | RUO | RUO | RUO | RUO | RUO |
| | | | | | | | | | | | | | | | | | |
| | | | | | 488 nm | 561 nm | | 638 nm | | 405 nm | | | | | | | |
| | | | | | 525/40 | 585/42 | 710/50 | 660/10 | 763/43 | 450/45 | | | | | | | |
| | | Biotin | | | FITC | PE | PC5.5 | APC | AFire750 | BV421 | | | | | | | |
| | | | | | | | | | | | | | | | | | |
| FIGURE 3A | Antigen | TCR γδ | TCR-Vα24 | TCR Vα7.2 | Biotin | CD161 | CD25 | CD56 | CD3 | CD4 | | | | | | | |
| | Clone | 11F2 | REA948 | REA179 | REA746 | HP-3G10 | M-A251 | HCD56 | SK7 | SK3 | | | | | | | |
| | Isotype | mIgG1 | rhuman IgG1 | rhuman IgG1 | rhuman IgG1 | mIgG1 | mIgG1 | mIgG1 | mIgG1 | rIgG1 | | | | | | | |
| | Fluorochrome | N/A | N/A | N/A | Vio Bright B515 | PE | PC5.5 | APC | APC-H7 | BV421 | | | | | | | |
| | Vol. (µl) in 100µl | 9 | 9 | 9 | 2 | 2 | 5 | 5 | 5 | 3 | | | | | | | |
| | Supplier | Miltenyi | Miltenyi | Miltenyi | Miltenyi | BioLegend | BioLegend | BioLegend | BD | BioLegend | | | | | | | |
| | Cat.# | 130-113-502 | 130-117-958 | 130-100-187 | 130-110-957 | 339904 | 356112 | 318310 | 560176 | 344632 | | | | | | | |
| | Status | RUO | RUO | RUO | RUO | RUO | RUO | RUO | RUO | RUO | | | | | | | |
| | | | | | | | | | | | | | | | | | |
| | | | | | 488 nm | 561 nm | | | 638 nm | | 405 nm | | 808 nm | | | | |
| | | | | | 525/40 | 585/42 | 710/50 | 763/43 | 660/10 | 763/43 | 525/40 | 610/20 | 885/40 | | | | |
| | | Biotin | | | FITC | PE | PC5.5 | PC7 | APC | AFire750 | BV510 | BV605 | Viability | | | | |
| | | | | | | | | | | | | | | | | | |
| FIGURE 3D | Antigen | TCR γδ | TCR-Vα24 | TCR Vα7.2 | Biotin | CD161 | CD25 | PLZF | FoxP3 | CD3 | CD127 | CD56 | Live/Dead | | | | |
| | Clone | 11F2 | REA948 | REA179 | REA746 | HP-3G10 | M-A251 | 9E12 | 259D | SK7 | A019D5 | HCD56 | | | | | |
| | Isotype | mIgG1 | rhuman IgG1 | rhuman IgG1 | rhuman IgG1 | mIgG1 | mIgG1 | HamsterIgG | mIgG1 | mIgG1 | mIgG1 | mIgG2 | | | | | |
| | Fluorochrome | N/A | N/A | N/A | Vio Bright B515 | PE | PC5.5 | PE/Cyanine7 | AF647 | APC-H7 | BV510 | BV605 | ViaKr-808 | | | | |
| | Vol. (µl) in 100µl | 9 | 9 | 9 | 2 | 2 | 5 | 2.5 | 2 | 5 | 2 | 5 | 2.5 | | | | |
| | Supplier | Miltenyi | Miltenyi | Miltenyi | Miltenyi | BioLegend | BioLegend | eBioscience | BioLegend | BD | BioLegend | BioLegend | BC | | | | |
| | Cat.# | 130-113-502 | 130-117-958 | 130-100-187 | 130-110-957 | 339904 | 356112 | 16340804 | 320213 | 560176 | 351332 | 318334 | C36628 | | | | |
| | Status | RUO | RUO | RUO | RUO | RUO | RUO | RUO | RUO | RUO | RUO | RUO | RUO | | | | |
Supplementary Figure 5. Flow cytometry panels to characterize T2NKT cells in PBMC (Figure 1A) and liver (Figure 1B), MACS-enriched sample (Figure 2C), FACS sorting (Figure 3A), and expanded cells (Figure 3D).
